# Development of Pacing, Electrophysiology and Defibrillation in India

**Published:** 2002-04-01

**Authors:** Mohan Nair, Johnson Francis, K Venugopal

**Affiliations:** 1 Director, Metro Golden Heart Institute, New Delhi, India; 2 Associate Professor of Cardiology, Medical College Calicut, Kerala, India; 3 Professor and Head, Department of Cardiology, Medical College Calicut, Kerala, India

## Cardiac Pacing

History of cardiac pacing in India dates back to late 1960s. Kar [1] reported that cardiac pacing was introduced in India in 1966. Basu [2] while discussing on cardiac pacemaking in Calcutta, mentions that the first pacing was performed in April 1967 at the Institute of Post Graduate Medical Education and Research (IPGME&R). Bhatia et al [3]  started pacemaker implantation at AIIMS, New Delhi in 1968. Their first patient was a doctor from Assam and the pulse generator was supplied by Medtronic Inc. The pulse generator was powered by a mercury-iodide battery which lasted for about 2 ½ years, after which the patient underwent pulse generator replacement. Unfortunately he succumbed to miliary tuberculosis about a year after that. Currently around 8000 pacemakers are being implanted annually in India, in various centers around the country.

The IPGME&R is the centre which implants maximum number of pacemakers in the country [1]. Various hypotheses has been put forward to explain the high incidence of conduction system disorders in the Eastern part of the country. Mustard oil is the usual cooking medium in the Eastern part of the country. Erucic acid content of Mustard oil is around 48%. Studies conducted by the Indian Council of Medical Research [4] has shown erucic acid content in all the 50 heart samples obtained from Calcutta (Eastern part of India) while none of the samples from Trivandrum or Madras (South India) showed any trace of erucic acid. The cooking medium in the latter regions is coconut oil, with no erucic acid content. Erucic acid has been shown to produce myocardial fibrosis and lipidosis [5-7]. High incidence of heart block has also been reported from Sweden [8] , where the cooking medium is rape-seed oil which also has a high erucic acid content. More studies are needed to conclusively prove whether Mustard oil is the etiological factor in the high incidence of heart block in the Eastern regions of India.

The first temporary pacemaker manufactured in India was the Khalilullah-Mendez pacemaker, which was marketed in 1970. It was a single chamber fixed rate pacemaker with facility for adjustment of pacing rate and pacing current. The device was jointly developed by Prof. M. Khalilullah at G.B. Pant Hospital, New Delhi and Mendez, an engineer-entrepreneur. Khalilullah-Mendez team also manufactured an indigenous monitor-defibrillator which was known as KM monitor-defibrillator.

**Figure 1:**
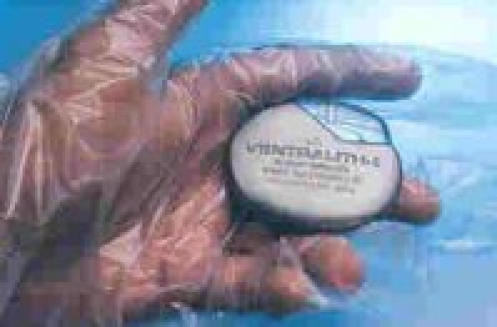
Ventralith-I : VVI Non Programmable Pacemaker

**Figure 2:**
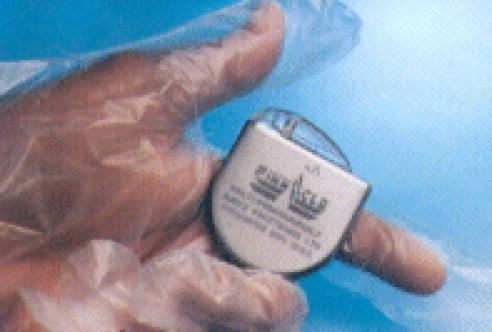
Pinnacle: VVI, multiprogrammable pacemaker

**Figure 3:**
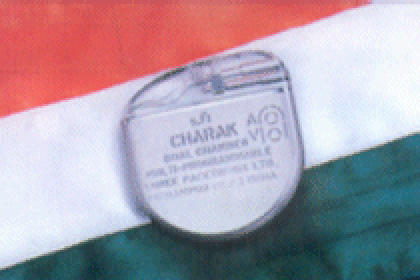
Charak: DDD, multiprogrammable pacemaker

The first permanent pacemaker manufactured in India was from Shree Pacetronix Ltd. The pulse generator was called Ventralith-I and it was a non-programmable VVI pacemaker. First implant was on September 9^th^, 1994 at Kastruba Hospital, Bhopal. Subsequently Shree Pacetronix has started manufacturing multiprogrammable pacemakers as well. The first multiprogrammable pacemaker was implanted on June 6^th^, 1995 at Ramakrishna Mission Seva Pratishthan, Calcutta. At present single lead VDD pacemakers, multiprogrammable DDD pacemakers (Figure 1-3) and single chamber pacing system analyzers are also being manufactured by Shree Pacetronix and they continue to be the only pacemaker manufacturer in India.

## Invasive Cardiac Electrophysiology

**Figure 4:**
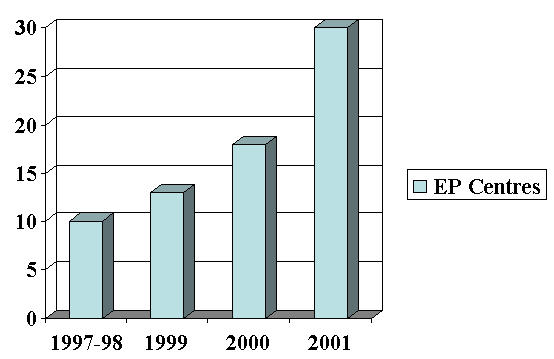
Number of EP Centres in India

**Figure 5:**
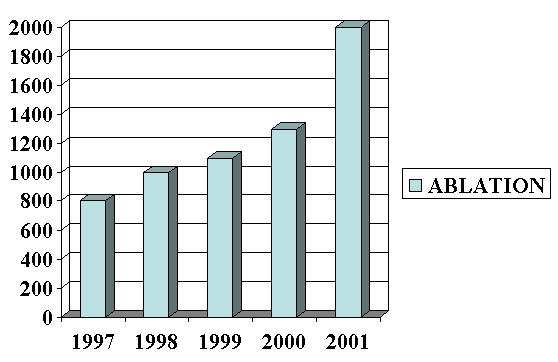
Number of RF Ablations in India

G.B. Pant Hospital New Delhi has been the pioneering center in the field of Invasive Cardiac Electrophysiology in the country. Bhatia M.L et al [9] and Khalilullah M et al [10-13] were the pioneers in His bundle electrography in the country. Prof. K.K. Sethi was the first to perform catheter ablation in the country. Direct Current ablation was started in 1988 followed by Radio Frequency (RF) ablation later on. The number of centres having electrophysiology setup in the country has increased from about 10 in 1997 to around 30 in 2001 (Figure 4). The number of RF ablations being performed in the country has gone up from 800 to 2000 during the same period (Figure 5). At least 3 government aided centers are offering subsidized diagnostic and therapeutic facilities. Average cost in private hospitals is around $ 200 for diagnostic procedures and $ 600 for ablation. Cost containment is mainly by frequent reuse of catheters. The national board has initiated post doctoral fellowship in electrophysiology. Three national and three regional electrophysiology workshops are being conducted every year in the country. Currently there are about 50 electrophysiologists under training.

## Research Activities in Electrophysiology

Newer insights in the field of atrial fibrillation in rheumatic heart disease have been obtained by electrophysiological studies from the country. Atrial disease due to rheumatic carditis is an important parameter, in addition to valvular involvement in the genesis of atrial fibrillation. Multiple substrates for atrial fibrillation are present in these cases, namely: stretch, fibrosis, raised pressures, increased atrial size and increased anisotropy. Atrial fibrillation in rheumatic heart disease is generally of long duration and affects a younger population.  Control of Rate versus Rhythm in Rheumatic Atrial Fibrillation Trial -'CRRAFT' conducted at Mumbai has documented the effectiveness of amiodarone in the treatment of rheumatic atrial fibrillation [14].  Mapping and successful RF ablation of flutter circuits seen at initiation after cardioversion of rheumatic atrial fibrillation has been achieved [15].

## Surgical RF Pulmonary Vein Isolation for Rheumatic Atrial Fibrillation

**Figure 6:**
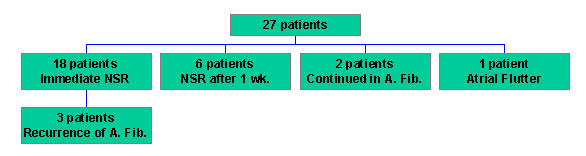
Surgical Pulmonary Vein Isolation: Indian Experience (Results)

Surgical RF pulmonary vein isolation using Thermaline Multi-Electrode Catheter [EP Technologies] has been done in 27 patients with rheumatic atrial fibrillation of more than one year duration. Lesions were as follows: bilateral pulmonary vein isolation, isolation of the left atrial appendage and connection lesion between left atrial appendage and pulmonary veins. The mean left atrial diameter was 7.2 cm. 18 patients regained sinus rhythm immediately while 6 patients achieved sinus rhythm after one week. Two patients continued to be in atrial fibrillation while one went into atrial flutter. Atrial fibrillation recurred in three of the successful cases. (Figure 6)

## Implantable Cardioverter

Prof. K.K. Talwar, AIIMS, New Delhi and Dr. T.S. Kler, Escorts Heart Institute & Research Centre, New Delhi were the first to implant Implantable Cardioverter Defibrillators (ICD) in India. Current annual ICD implantation rates stand at 60 per year.
